# Accuracy of Ultrasound Diagnosis of Benign and Malignant Thyroid Nodules: A Systematic Review and Meta-Analysis

**DOI:** 10.1155/2022/5056082

**Published:** 2022-09-13

**Authors:** Mei Shi, Dandan Nong, Minhui Xin, Lifei Lin

**Affiliations:** ^1^Department of Ultrasonic Medicine, Central South University Xiangya School of Medicine Affiliated Haikou Hospital, Haikou 570208, China; ^2^Department of Ultrasonic Medicine, Baisha Li Autonomous County People's Hospital, Baisha 572800, China; ^3^Department of Ultrasonic Medicine, Sanya Women and Children's Hospital Managed by Shanghai Children's Medical Center, Sanya 572000, China

## Abstract

**Background:**

Distinguishing between benign and malignant thyroid nodules remains difficult. Ultrasound has been established as a non-invasive and relatively simple imaging technique for thyroid nodules. This study aimed to assess the diagnostic accuracy of conventional ultrasound and ultrasound elastography for the differentiation between benign and malignant thyroid nodules by meta-analyzing published studies.

**Methods:**

Literature was retrieved from the PubMed and Embase databases from inception to May 31, 2022. The literature was screened using inclusion and exclusion criteria. The Quality Assessment of Diagnostic Accuracy Studies (QUADAS2) scale was used to assess the quality of the included literature. Publication bias of the included studies was assessed by Deek's funnel plot. Heterogeneity tests were performed using Cochrane *Q* statistic and I^2^ statistic.

**Results:**

Finally, 9 articles were included. The meta-analysis showed that the combined sensitivity and specificity of ultrasound for the diagnosis of thyroid nodules were 0.88 [95% CI (0.83–0.91)] and 0.86 [95% CI (0.79–0.90)], respectively. The area under the curve (AUC) of the summary receiver operating characteristic curve (SROC) was 0.92 [95% CI (0.90–0.94)]. There was no significant publication bias in this study. *Discussion*. Existing evidence shows that ultrasound has a certain accuracy in diagnosing benign and malignant thyroid nodules, providing a scientific basis for thyroid assessment and diagnosis.

## 1. Introduction

Thyroid nodules are cystic or solid lumps that are most frequently asymptomatic. Nonetheless, large thyroid nodules have also been shown to interfere with the normal functioning of cardiovascular and respiratory functions [1, 2]. Pathologically, thyroid nodules are dichotomized into benign nodules and malignant nodules. In general, most of the benign thyroid nodules are small in size, mild in symptoms, and have favorable treatment outcomes. Therefore, accurate and effective determination of the nature of nodules is beneficial for clinical treatment planning and assessment of outcomes [3–6]. Currently, the clinical techniques used to distinguish benign and malignant thyroid nodules mainly include ultrasound, computed tomography, and nuclear imaging. Traditional ultrasonography is widely used in clinical practice due to its advantages of safety, low cost, ready availability, and no radiation exposure [7–9]. The ultrasound images of malignant thyroid nodules have the characteristics of irregular shape, unclear edge, inhomogeneous, calcification, low echo, and aspect ratio greater than 1.” However, conventional ultrasound is limited for the diagnosis of malignant thyroid nodules in terms of small thyroid cancers, multiple nodules, and cystic nodules with internal hemorrhage. In addition, there are some thyroid nodules that are not obvious on ultrasound imaging. Thus, several studies have concluded that traditional ultrasound imaging techniques cannot actually meet the needs of current clinical practice [10–12].

Ultrasound elastography, a newly developed dynamic imaging technique, was first proposed by Ophir et al. in 1991 [13] and first applied to thyroid clinical practice by Lyshchik et al. in 2005 [14]. Subsequently, in 2010, Sebag et al. first reported the use of shear-wave elastography (SWE) to diagnose thyroid nodules [15]. In recent years, emerging studies have shown that ultrasound elastography is highly sensitive for differentiation between benign and malignant thyroid nodules and should serve as the first-line imaging modality for patients with thyroid nodule [16, 17].

Therefore, this study evaluated the diagnostic accuracy of conventional ultrasound and ultrasound elastography for the differentiation between benign and malignant thyroid nodules by meta-analyzing published studies.

## 2. Methods

### 2.1. Literature Source

Electronic databases, including PubMed and Embase, were searched from inception to May 31, 2022. Keywords used for searching included ultrasonography and thyroid nodule. The combination of medical subject headings and free words was used to search relevant publications. The retrieved literature was checked manually and managed by EndNote X9.

### 2.2. Inclusion and Exclusion Criteria of Literature

Studies that meet the following criteria were included: (1) the study evaluated the diagnostic utility of conventional ultrasound or ultrasound elastography for patients with thyroid nodules; (2) pathological biopsy was used as the “gold standard” for determination of the benignity or malignancy of the thyroid nodule; and (3) research could directly or indirectly obtain true positive, false positive, false negative, and true negative value. The exclusion criteria were as follows: (1) guidelines, reviews, meetings, reviews, meta-analysis, and other non-original articles; (2) repeated publication; and (3) incomplete data.

### 2.3. Literature Screening, Data Extraction, and Quality Evaluation

Literature retrieval, screening, and data extraction were completed by two researchers independently. Two researchers made standardized tables to extract data from the included literature, including research author, research time, country, and type of experiment. The patient data were recorded, including the total number of cases, diagnostic reference standards, and the number of thyroid nodules. The number of true positive, false positive, true negative, and false negative was also extracted from the included studies. The quality of the included literature was evaluated by the Quality Assessment of Diagnostic Accuracy Studies (QUADAS2) scale. Two researchers cross-checked the quality assessment results. If there is any disagreement, the joint judgment result after consultation and discussion shall prevail.

### 2.4. Statistical Analysis

Stata V 15.0 software was used for statistical analysis. The combined effect quantity, including sensitivity, specificity, positive likelihood ratio, negative likelihood ratio, and diagnostic odds ratio, was obtained. The diagnostic capability was evaluated by drawing the subject operating characteristic curve (SROC). A larger area under the curve (AUC) often signified higher diagnostic accuracy. Heterogeneity test was performed using I^2^. In the included literature, *P* < 0.05 or I^2^ > 50% indicated high heterogeneity; *P* > 0.1 or I^2^ < 25% indicated low heterogeneity; and 25% ≤ I^2^ ≤ 50% indicated moderate heterogeneity. If the inter-study heterogeneity is high, the random-effects model is used for meta-analysis; otherwise, a fixed-effect model is used for meta-analysis. Publication bias detection was performed using Deek's funnel plots. Two-sided *P* value < 0.05 denoted statistical significance.

## 3. Results

### 3.1. Literature Search Results

**Figure 1:**
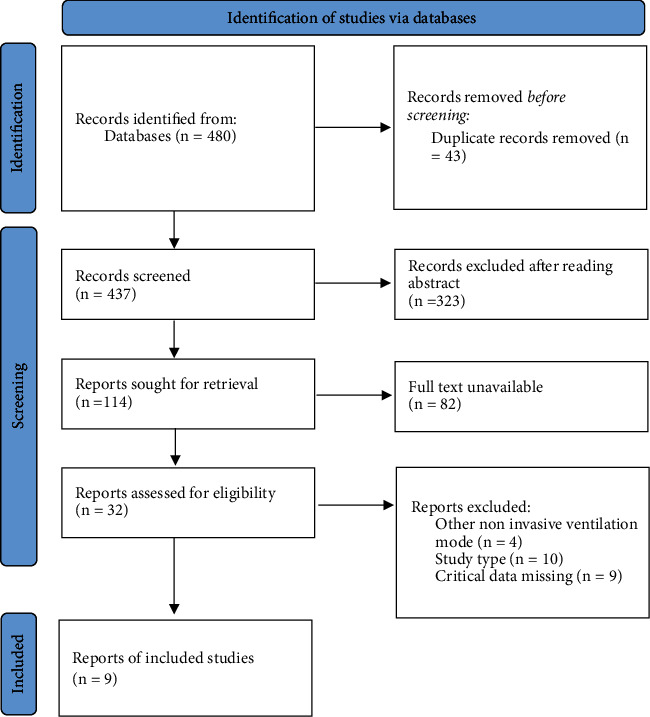
Flowchart of literature screening. The process of screening meta-analysis into the literature.

After the preliminary search, 480 studies were retrieved. According to the inclusion and exclusion criteria, 43 duplicate studies were excluded. After reading the title and abstract, 323 obviously unrelated studies were excluded. A total of 32 publications were downloaded and read for the full text. Finally, 9 studies were included, as shown in Figure 1.

### 3.2. Basic Characteristics of Included Articles

All the 9 included articles were English publications that included 7 prospective single-center studies, 1 prospective multicenter study, and 1 retrospective study. A total of 1436 nodules were included, including 1006 benign nodules and 430 malignant nodules, as shown in Table 1.

### 3.3. Quality Evaluation of Included Studies

**Figure 2:**
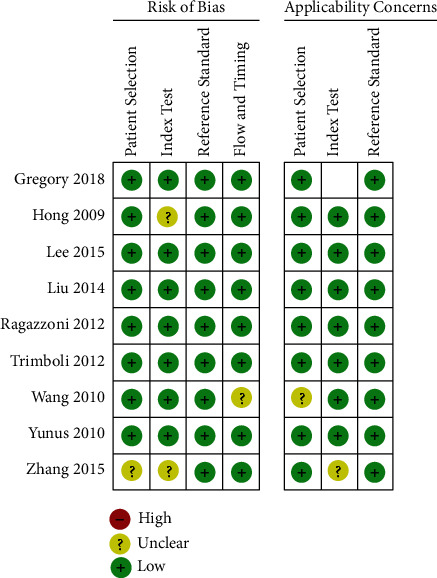
Quality evaluation graph of included studies. Green represents low risk of bias.

QUADAS2 scale was used to evaluate the quality of the 9 included articles (Figure 2). The articles we included were all of low risk.

### 3.4. The Results of Meta-Analysis

#### 3.4.1. Heterogeneity Test

**Figure 3:**
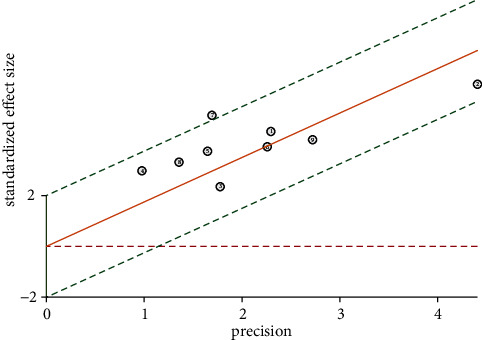
Heterogeneity test for ultrasound diagnosis of thyroid nodules.

All included studies were tested for heterogeneity. There was significant inter-study heterogeneity (I^2^ = 70%) (Figure 3), so the random-effects model was used for pooled analysis.

#### 3.4.2. Consolidation Analysis

**Figure 4:**
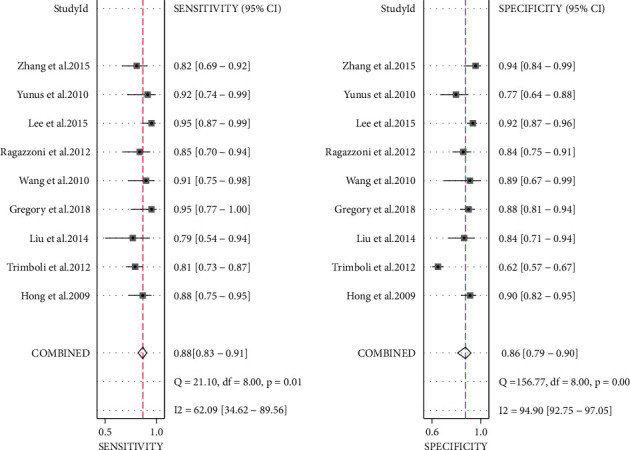
The forest plot of sensitivity and specificity in thyroid nodule ultrasound diagnosis.

**Figure 5:**
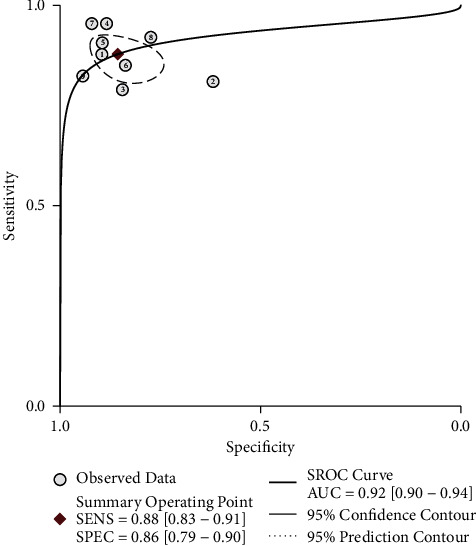
Subject operating characteristic curve (SROC) for ultrasound diagnosis of thyroid nodules.

**Figure 6:**
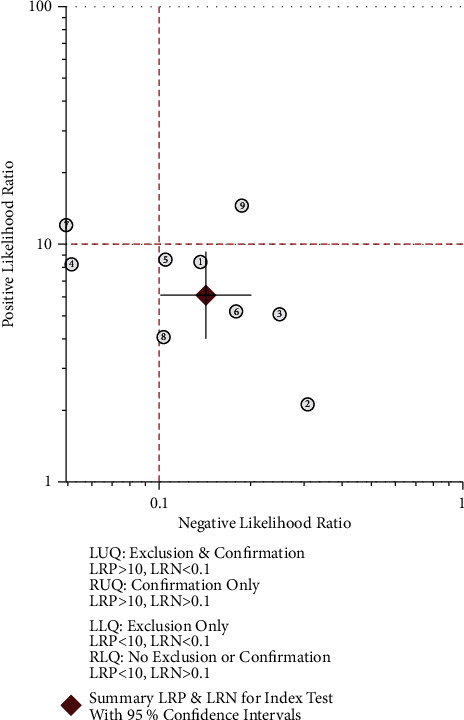
Dot plot of likelihood ratio for ultrasound diagnosis of thyroid nodules.

**Figure 7:**
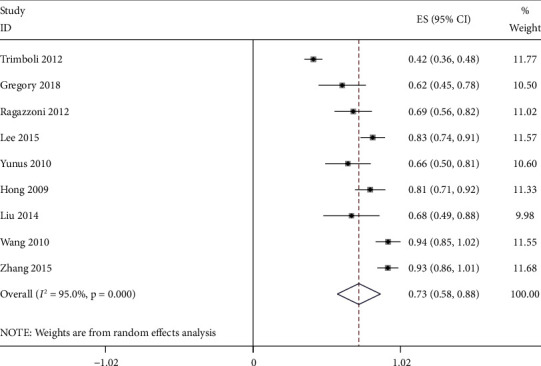
Positive likelihood ratio for ultrasound diagnosis of thyroid nodules.

**Figure 8:**
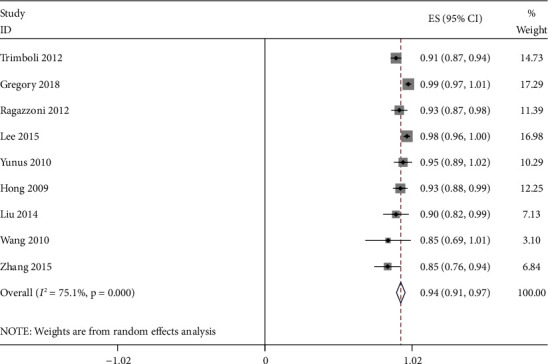
Negative likelihood ratio for ultrasound diagnosis of thyroid nodules.

The effect quantities of all included studies were statistically analyzed. The combined sensitivity and specificity were 0.88 [95% CI (0.83–0.91)] and 0.86 [95% CI (0.79–0.90)], respectively. The combined positive and negative likelihood ratio was 0.73 [95% CI (0.58–0.88)] and 0.94 [95% CI (0.91–0.97)], respectively (Figures 4–8). The AUC under SROC was 0.92 [95% CI (0.90–0.94)] (Figures 4–8).

#### 3.4.3. Fagan Nomogram Analysis

**Figure 9:**
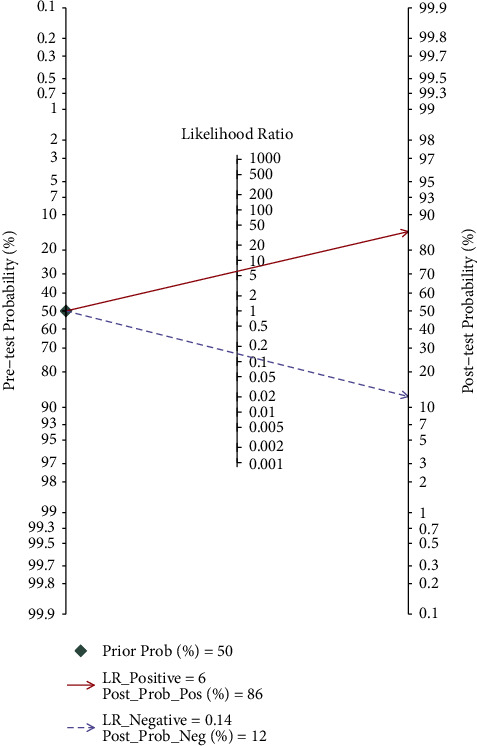
Fagan graph of the accuracy of ultrasonography in diagnosing benign and malignant thyroid nodules.

A 50% predicted probability was used to simulate the clinical situation. The results showed that the post-test probability of a positive test result was 86%, while the negative likelihood ratio was 0.14 and the negative post-test probability was 1% (Figure 9).

### 3.5. Meta-Regression and Subgroup Analysis

**Figure 10:**
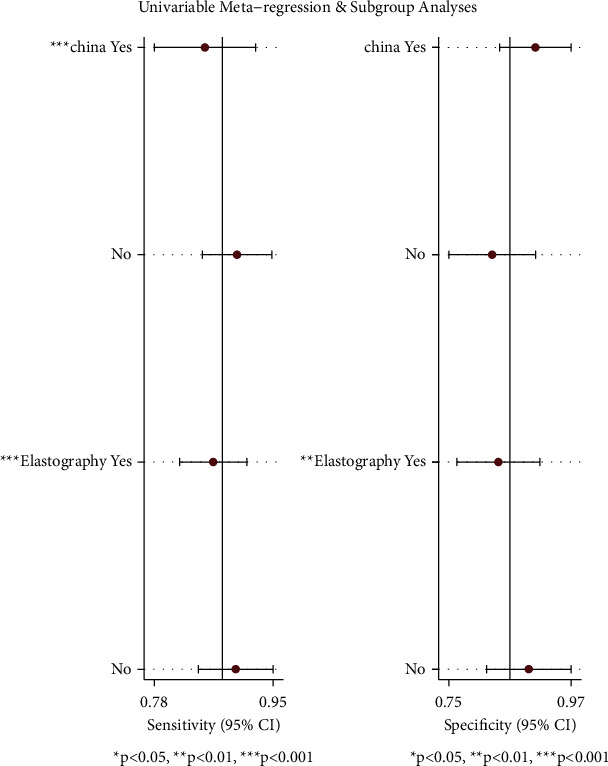
Univariable meta-regression and subgroup analyses of the accuracy of ultrasound in the diagnosis of benign and malignant thyroid nodules.

There was no significant difference in specificity between articles from China and those that are not (*P*=0.28). Sensitivity was significantly different between studies in the Chinese group at 0.85 [95% CI (0.78–0.93)] and in the non-Chinese group at 0.90 [95% CI (0.85–0.95)]. The diagnostic sensitivity and specificity of ultrasound elastography were 0.86 [95% CI (0.82, 0.91)] and 0.84 [95% CI (0.76–0.91)], respectively. The sensitivity and specificity of conventional ultrasound diagnosis were 0.84 [95% CI (0.76–0.91)] and 0.89 [95% CI (0.81–0.97)], respectively. There were significant differences in terms of both the sensitivity and specificity (*P* < 0.05). Diagnosis was a potential factor for heterogeneity. The results are shown in Table 2 and Figure 10.

### 3.6. Publication Bias

**Figure 11:**
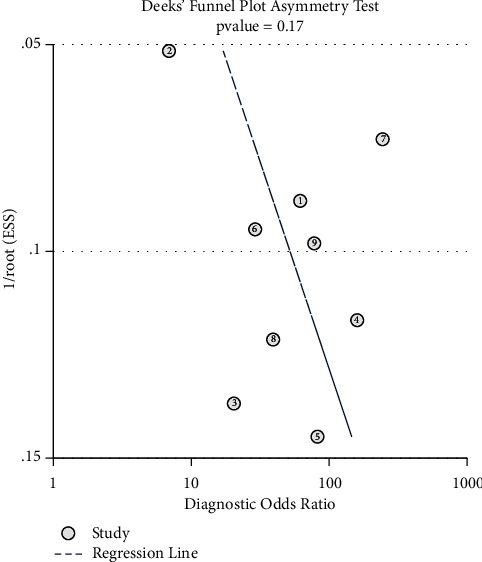
Publication bias detected by using Deek's funnel plot.

The results of publication bias detection are shown in Figure 11. The *P* value for the slope coefficient of Deek's funnel plot is 0.17, indicating no significant publication bias in the included studies.

## 4. Discussion

According to the inclusion criteria, 9 research articles with 1436 thyroid nodules from 1378 patients were selected to analyze the ultrasonic differentiation of benign and malignant thyroid nodules. Since high heterogeneity was observed in the analysis results, the random-effects model was applied in the data analysis. The sensitivity and specificity of ultrasound diagnosis were 0.88 [95% CI (0.83–0.91)] and 0.86 [95% CI (0.79–0.90)], respectively. The combined positive and negative likelihood ratio was 0.73 [95% CI (0.58–0.88)] and 0.94 [95% CI (0.91–0.97)], respectively. Furthermore, the area under the SROC curve of ultrasound diagnosis of thyroid nodules was 0.92 [95% CI (0.90–0.94)]. Therefore, the result demonstrated a good diagnostic efficiency. At the same time, the results of subgroup analysis showed that the diagnostic sensitivity and specificity of ultrasound elastography were 0.86 [95% CI (0.82, 0.91)] and 0.84 [95% CI (0.76–0.91)], respectively. The diagnostic sensitivity and specificity of conventional ultrasound were 0.84 [95% CI (0.76–0.91)] and 0.89 [95% CI (0.81–0.97)], respectively. In addition, there are significant differences in terms of diagnostic sensitivity and specificity between ultrasound elastography and conventional ultrasound, indicating that the diagnosis method may be a potential factor of heterogeneity.

Due to its non-invasiveness, wide availability, and low cost, ultrasonography is still the preferred method for clinical examination of thyroid nodules. In recent years, the Thyroid Imaging Reporting and Data System (TIRADS) risk score has been introduced clinically to standardize the risk assessment of ultrasonographic diagnosis of malignant thyroid nodules [27–30]. The main advantage of the TIRADS score is its high accuracy for identifying suspicious thyroid nodules worthy of cytological examination, thereby achieving early detection while avoiding unnecessary biopsies [31, 32]. However, TIRADS also has some limitations in practical applications in recent years. For instance, thyroid nodules of different classifications may have the same TIRADS score. A study from Italy in 2017 showed that the accuracy of the TIRADS score was approximately 27.2% [33]. In contrast, studies have shown that the specificity and sensitivity of fine-needle aspiration (FNA) in identifying malignant thyroid nodules were about 60%–98% and 54%–90%, respectively. FNA remains one of the gold standards for identifying malignant thyroid nodules [34–37].

Ultrasound evaluation of the lateral neck during the early assessment is helpful in determining the scope of the final operation [38]. Some studies have found that preoperative neck ultrasound has changed the surgical method in 40% of patients [38–40]. At this stage, it is recommended that all patients with suspected thyroid nodules should undergo an ultrasound examination [16]. Hyperechoic/isoechoic (brighter than normal thyroid tissue or with the same echo) nodules are usually benign. Meanwhile, noticeable hypoechoic nodules increase the risk of malignancy [41, 42]. Nodules with mixed cystic and solid components are less likely to be malignant than completely solid nodules [43, 44]. “Taller-than-wide” appearance also increases the risk of malignancy [45, 46]. Intra-nodal calcification has also been reported to increase the likelihood of malignancy [47, 48]. A study of nearly 700 thyroid tumors found that more than half of malignant nodules (63%) lacked intra-nodal vessels on preoperative imaging [49].

Various cancerous processes alter the physical characteristics of affected tissues. Ultrasound sonography is a novel imaging technique that can provide information about tissue hardness [14, 50–55]. With emergence of commercial ultrasound systems, ultrasound elastography has been increasingly applied in various fields to verify its clinical applicability [51, 52, 56–59]. Among the 40 patients examined with ultrasound elastography, 35 of the 40 benign nodules and 9 of the 11 malignant nodules have been correctly classified by ultrasound elastography with pathological examination as the reference standard [60].

The assessment and management of patients with thyroid nodules is no longer a one-size-fits-all proposition. The main challenge in the management of thyroid nodules is to identify malignant nodules while avoiding excessive use of aspirations and surgery at the same time. Therefore, advanced diagnostic methods that can accurately evaluate the benign and malignant thyroid nodules would be desirable. A customized method is advocated, which requires careful evaluation of each nodule to determine the possibility of malignancy [1]. Ultrasound can maximize the detection of clinically relevant thyroid lesions and reduce fine-needle aspiration of benign nodules to reduce over-diagnosis and over-treatment of benign nodules, achieving the best prognosis for patients and minimizing the cost of medical treatment [61].

The 9 studies included in this study have some heterogeneity after analysis, which might affect the reliability of the study conclusions to a certain extent. We suspected that possible reasons for high inter-study heterogeneity were related to small sample size and incomplete publication inclusion since databases other than PubMed and Embase were not searched. In addition, the experience of ultrasound operators would also affect the study results.

In conclusion, ultrasound is still an ideal way to detect thyroid nodules. In the future, additional research is required to improve ultrasonic diagnosis. Meanwhile, it can be combined with other relevant imaging technologies to improve the sensitivity and specificity of ultrasonic diagnosis and reduce unnecessary pathological aspirations. Furthermore, the diagnostic accuracy can be improved by fine-needle aspiration biopsy and other imaging examinations if the lesions are not determined by routine ultrasound.

## Figures and Tables

**Table 1:** The study's basic characteristics and quality score.

Included studies	Country	TP	FP	FN	TN	Type of study
Hong et al. [18]	China	43	10	6	86	Prospective study
Trimboli et al. [19]	Italy	102	142	24	230	Prospective multicenter study
Liu et al. [20]	China	15	7	4	38	Prospective study
Gregory et al. [21]	United States	21	13	1	99	Prospective study
Wang et al. [22]	China	29	2	3	17	Prospective study
Ragazzoni et al. [23]	Italy	34	15	6	77	Prospective study
Lee et al. [24]	Korea	63	13	3	151	Retrospective study
Yunus et al. [25]	Pakistan	23	12	2	41	Prospective study
Zhang et al. [26]	China	42	3	9	50	Prospective study

**Table 2:** Univariable meta-regression.

Parameter	Category	N	Sensitivity	P1	Specificity	P2
China	Yes	4	0.85 (0.78–0.93)	<0.001	0.90 (0.84–0.97)	0.28
	No	5	0.90 (0.85–0.95)	—	0.82 (0.75–0.90)	—
US-Elastography	Yes	6	0.86 (0.82, 0.91)	<0.001	0.84 (0.76–0.91)	<0.01
	No	3	0.84 (0.76–0.91)		0.89 (0.81–0.97)	—

## Data Availability

The data used and analyzed during the current study are available from the corresponding author upon request.
